# C-Phycocyanin Ameliorates the Senescence of Mesenchymal Stem Cells through ZDHHC5-Mediated Autophagy *via* PI3K/AKT/mTOR Pathway

**DOI:** 10.14336/AD.2023.0121

**Published:** 2023-08-01

**Authors:** Guoxiang Liu, Xiaoxia Li, Fanghao Yang, Jingyu Qi, Lipeng Shang, Huhu Zhang, Shuang Li, Fenghua Xu, Lingne Li, Huaxin Yu, Yang Li, Xiaolei Dong, Qinghang Song, Feng Zhu, Guang Chen, Can Cao, Liangqian Jiang, Junzhe Su, Lina Yang, Xiaohui Xu, Zhe Zhang, Robert Chunhua Zhao, Bing Li

**Affiliations:** ^1^Department of Genetics and Cell Biology, Basic Medical College, Qingdao University, Qingdao, China.; ^2^Department of Medical Genetics, Linyi People's Hospital, Linyi, China.; ^3^College of Basic Medicine, Institute of Stem Cell and Regenerative Medicine, Qingdao University, Qingdao, China.; ^4^Institute of Basic Medical Sciences Chinese Academy of Medical Sciences, School of Basic Medicine Peking Union Medical College, Beijing, China.; ^5^School of Life Sciences, Shanghai University, Shanghai, China.; ^6^Department of Hematology, The Affiliated Hospital of Qingdao University, Qingdao, China

**Keywords:** MSCs, Senescence, C-PC, ZDHHC5, Autophagy

## Abstract

The senescence of mesenchymal stem cells (MSCs) impairs their regenerative capacity to maintain tissue homeostasis. Numerous studies are focusing on the interventions and mechanisms to attenuate the senescence of MSCs. C-phycocyanin (C-PC) is reported to have multiple functions such as antitumor, antioxidation, anti-inflammation and anti-aging roles, but there is little research about the effects of C-PC on the senescence of MSCs. Here we investigated the roles and mechanism of C-PC on MSCs senescence. *In vitro* results showed that C-PC could reduce senescence, enhance proliferation, promote the adipogenic and osteogenic differentiation in senescent MSCs induced by oxidative stress. *In vivo* D-Galactose (D-Gal) induced rats aging models showed C-PC also increased the viability and differentiation of intrinsic senescent bone marrow derived MSCs (BMSCs). Furthermore, C-PC also decreased the levels of oxidative stress markers ROS or MDA, elevated the SOD activity, and increased the anti-inflammatory factors. Proteomic chip analysis showed that C-PC interacted with ZDHHC5, and their interaction was verified by pull down assay. Overexpression of ZDHHC5 aggravated the senescence of MSCs and greatly lessened the beneficial effects of C-PC on senescence. In addition, we found ZDHHC5 regulated autophagy by altering LC3, Beclin1 and PI3K/AKT/mTOR pathway. In summary, our data indicated that C-PC ameliorates the senescence of MSCs through zinc finger Asp-His-His-Cys (DHHC) domain-containing protein 5 (ZDHHC5) mediated autophagy via PI3K/AKT/mTOR pathway. The present study uncovered the key role of autophagy in MSCs senescence and PI3K/AKT/mTOR pathway may be a potential target for anti-senescence studies of MSCs.

## INTRODUCTION

Aging is the biological process of gradual decline in physiological functions over time. The cellular and molecular hallmarks of aging process include genomic instability, telomere attrition, epigenetic alterations, loss of proteostasis, deregulated nutrient-sensing, mitochondrial dysfunction, cellular senescence, stem cell exhaustion, and altered intercellular communication [1]. Cell senescence is the cellular basis of biological aging, which leads to the development and progression of aging-related diseases such as cancer, diabetes, cardiovascular disorders and neurodegenerative diseases [2-5]. Nowadays the investigation and intervention of cell senescence have attracted more and more attention.

The senescence and exhaustion of stem cells are considered important drivers of organism aging [6]. Stem cell senescence is a cellular response to endogenous or exogenous stresses [7]. Adult stem and progenitor cells are responsible for tissue maintenance, repair, and regeneration. During the process of aging, this population of cells is decreased, and their activities are reduced which compromise the tissue integrity and cause pathologies [8]. Age-associated stem cell senescence has been confirmed as an important factor for the pathogenesis of multiple disorders. For example, hematopoietic stem cells (HSCs) gradually become senescent with aging, lose their self-renewal and regenerative potential, and weaken the adaptive immune system [9]. BMSCs aging may contribute to aging-related osteoporosis [10]. BMSCs extracted from osteoporosis patients acquired several abnormalities including reduced proliferation and osteogenic differentiation potential. Therefore BMSCs senescence is regarded as a major driver in the pathogenesis of age-related bone loss [11].

MSCs are tissue-specific progenitor cells with self-renewal and multi-potent differentiation potentials. They were found not only in bone marrows, but also in various tissues like adipose tissue, skeletal muscle, umbilical cord blood, placenta, and dental pulp. MSCs can differentiate into several different cell types including osteocytes, chondrocytes, adipocytes and so on [7, 12]. MSCs can automatically migrate toward injury areas and spontaneously differentiate into desired tissues to perform regenerative functions [12]. However, the regenerative capacity of MSCs to maintain tissue homeostasis declines progressively and the senescence of MSCs increases with aging [13-15]. Senescent MSCs not only hamper tissue repair but also mediate tissue degeneration by initiating and spreading senescence-associated inflammation [7].

In order to maintain MSCs from being senescent, pharmacological interventions are being explored [1]. For example, ascorbic acid could inhibit the senescence of MSCs through ROS and Akt/mTOR signaling [16]. Trans-cinnamaldehyde (TC) treatment reduced the senescence of the adipose derived MSCs (AMSCs) induced by H_2_O_2_ and restored their functionality *in vivo* [17]. Likewise, many other small molecular compounds such as quercetin, rapamycin, resveratrol and melatonin are reported to inhibit stem cell senescence [18].

C-PC is a natural active protein extracted from *Spirulina platensis* which is composed of α and β subunits. Recent studies showed that C-PC has pharmaceutical potentials in anticancer [19], anti-inflammation [20] and antioxidation [21]. For example, Zhang et al. found that C-PC could elicit antitumor efficacy via causing cell-cycle arrest and apoptosis of esophageal squamous cell carcinoma [22]. Ji et al. reported C-PC inhibited epithelial-to-mesenchymal transition (EMT) in caski cervical cancer cells [23]. Besides, C-PC has been shown to be effective in improving tissue and organ inflammation including osteoarthritis, colitis etc. [24, 25]. In particular, C-PC could serve as a promising reproductive system protective agent against low fertility by inhibiting ROS in D-Gal-induced aging mice [26, 27] . Similarly, C-PC also prevents acute myocardial infarction-induced oxidative stress, inflammation, and cardiac damage [28]. Whether C-PC could be applied against MSCs senescence is not known. Herein, we evaluated the effects of C-PC on oxidative stress-induced MSCs senescence *in vitro* and D-Gal-induced aging rat model *in vivo*. Moreover, we investigated the underlying molecular mechanism by using proteome chip and transcriptome sequencing. Our results suggested that C-PC could act as an anti-senescence agent via ZDHHC5 mediated PI3K/AKT/mTOR pathway to induce autophagy and finally prevent MSCs senescence induced by oxidative stress.

## MATERIALS AND METHODS

### Extraction, cultivation, and identification of primary MSCs

Primary MSCs were obtained by separating adipose tissue discarded from normal people after liposuction as reported previously[29]. All experiments were approved by the Qingdao University Ethics Committee. After collagenase (Sigma Aldrich, USA) digestion, purified MSCs were cultured in DME/F-12 complete medium (HyClone, USA) supplemented with fetal bovine serum (ExCell Bio, Shanghai, China), penicillin-streptomycin (New cell& Molecular Biotech, Suzhou, China)and incubated in a humid incubator at 37°C and 5% CO_2_. At the fourth passage, MSCs were collected and incubated with different phenotypic antibodies at 4°C for 30min (For intracellular molecules, MSCs were fixed with 4% paraformaldehyde at room temperature, and then ruptured with 0.1% Triton X-100 solution). The manufacturer and catalog of antibodies we used were listed in Supplement Table. After incubation, the cells were washed and re-suspended. Analysis was performed on Accuri C6 Flow Cytometers with CFlow software (Accuri Cytometers, Inc.).

### Cell Counting Kit-8 Assay

Cells were seeded in 96-well plates (5000/well) and cultured with different concentrations of hydrogen peroxide solution reagent (3%) (Sigma Aldrich, USA) and C-PC (Binmei Biotechnology, Taizhou, China) for appropriate time. After incubation, the drugs were removed and then 100 μl of CCK-8 mixture (1:10) (Biosharp, Beijing, China) was added. The cells were incubated in a cell incubator for an additional 2 h and the absorbance at 450 nm was measured.

### Senescence β-Galactosidase Stain

Cells were cultured in 6-well plates and washed with PBS after drug treatment. Then 1 ml of β-galactosidase staining fixative (Solarbio, Beijing, China) was added for 15 min at room temperature. After removal of the fixative, the cells were washed 3 times with Phosphate Buffered Saline (PBS) (Biosharp, Beijing, China) for 3 min each. PBS was aspirated and 1 ml of staining working solution was added to each well. Preparation method of staining solution: 10 μl of β-galactosidase staining solution A, 10 μl of β-galactosidase staining solution B, 930 μl of β-galactosidase staining solution C and 50 μl of X-Gal solution. Cells are incubated overnight at 37°C and 6-well plates were covered with plastic wrap to prevent evaporation. Cells were observed and counted under a common optical microscope. Green indicates senescent cells. The percentage of senescence (%) was calculated by counting the number of stained cells and dividing them into the total number of cells.

### Reactive Oxygen Species Assay

The fluorescent probe DCFH-DA (1:1000) (Beyotime Biotechnology, Beijing, China) was diluted with serum-free medium to a final concentration of 10 μM. The original liquid in each well of the six-well plate was removed and 1ml of DCFH-DA diluent was added. The incubation lasted for 20min in a 37ºC cell incubator. Then the cells were washed three times with serum-free cell culture medium to sufficiently remove DCFH-DA that did not enter the cells. Finally, the cells were observed under a fluorescence microscope.

### SOD activity assay

SOD activity was determined by xanthine oxidase method (hydroxylamine). MSCs were lysed by RIPA lysis buffer and the supernatant was collected by centrifugation. The samples were prepared according to the manufacturer’s instructions of SOD Test Kit (Nanjing Jiancheng, Bioengineering Institute, Nanjing, China). The maximum absorbance at 550 nm was measured to calculate the SOD activity [30].

### MDA assay

The concentration of MDA was used to indicate lipid peroxidation. BMSCs were lysed by RIPA lysis buffer and the supernatant was collected by centrifugation. The samples were prepared according to the manufacturer’s instructions of MDA Test Kit (Nanjing Jiancheng, Bioengineering Institute, Nanjing, China). The concentrations of MDA were calculated by measuring the maximal absorbance at 532 nm [30].

### Adipogenic differentiation

The 4^th^ passage of AMSCs or BMSCs were seeded into 6-well plates and cultured to 80% confluence at 37°C, 5% CO_2_ cell incubator. The adipogenic induction medium was composed of 100 μg/ml isobutyl methylxanthine, 1 μM dexamethasone, 50 μg/ml ascorbate acid, 100 IU penicillin, 100 μg/ml streptomycin (Sigma Aldrich, USA) and DMEM high glucose medium (HyClone, USA) containing 10% FBS. During the induction, the adipogenic induction medium was changed every 3 days. On the 12th day, total protein was extracted to detect the expression of PPAR-γ. In addition, lipid droplets were detected by Oil Red O staining.

### Oil red O staining

Cells were washed twice with PBS and fixed with 4% paraformaldehyde for 10 min at room temperature. Then, the cells were washed three times with PBS and stained with filtered oil red O solution (Sigma-Aldrich, USA) (stock solution: 3 mg/ml in isopropanol; working solution: 60% oil red O stock solution and 40% distilled water) for 30 min at 37 °C. After staining, the cells were washed under flowing water for 1 min and then visualized and imaged by light microscopy.

### Osteogenic differentiation

The 4th passage of AMSCs or BMSCs were seeded into 6-well plates and cultured to 80% confluence at 37°C, 5% CO_2_ cell incubator. The components of osteogenic differentiation induction medium included 10mM β-glycerophosphate sodium, 100nM dexamethasone, 50 µM ascorbate acid, 100 IU penicillin, 100 μg/ml streptomycin (Sigma Aldrich, USA) and DMEM high glucose medium (HyClone, USA) containing 10% FBS. The osteogenic induction medium was changed every 3 days. On the 12th day, proteins were extracted to detect the expression of RUNX-2. The calcium mineralization deposition was detected by Alizarin Red S staining.

### Alizarin Red S staining

Cells in culture were washed twice with PBS, fixed in 4% paraformaldehyde for 10 min, and then washed with distilled water and stained with 1% Alizarin red (Leagene, Beijing, China) for 30 min at room temperature. Then, cells were washed with distilled water to remove the unbound dye, and calcification was visualized as red deposits under light microscopy.

### Proteome chip

HuProt™ arrays covering approximately 20,000 full-length human proteins were purchased from CDI Laboratories Inc. (CDI, USA). Each protein was printed in duplicate onto Hydrogel Shot slides which was immobilized with GST tag antibody. To identify potential interacting proteins, purified C-PC proteins were probed to the HuProt arrays. Briefly, the chip was stored at -80 °C and transferred to -20 °C. Then it was placed at 4 °C for 30 min. Next it was immersed in a precooled blocking solution (5% BSA/1×PBS-T) in a dish on a shaker at 50 rpm and blocked for 5 min at room temperature. The blocking solution was replaced, and the blocking continued for 1.5 h. After a brief wash with 1×PBS-T for 5 min, the chip was placed in a hybridization chamber containing C-PC proteins diluted in blocking buffer and the incubation lasted for 1 h at room temperature. After washing with 1×PBS-T solution and deionized water, the chip was dried by centrifugation at 1000 rpm for 2 min. Finally, the chip was scanned with a microarray scanner (GenePix 4000B). All procedures were in dark and each experiment was performed in triplicates [31].

### GST pull down assay

GST-V, GST-C-PC α, GST-C-PC β proteins were expressed and purified as reported previously and adjusted to 5 μg/μl as the final concentration [32, 33]. AMSCs (3×10^7^) cells were lysed with 2ml RIPA lysis buffer, and 200 μl was used as input. The remaining lysates were incubated with 30 μg of GST-V beads at 4°C for 1 h. After co-incubating, the samples were divided into 3 equal volumes and incubated overnight with 30 μg of GST-V, GST-α, GST-β beads (Sangon Biotech,China) on a shaker at 4°C. Then, the samples were centrifuged at 2000 rpm, 4 °C for 2 min. The supernatant was discarded, and the pre-prepared magnetic bead wash was used for gradient washing. Finally, the proteins were further detected by western blot.

### ZDHHC5 overexpression

AMSCs were transfected with ZDHHC5 expressing lentivirus using HiTransG A (Shanghai Genechem, Shanghai, China) for 72 h. Following infection, the cells were selected with puromycin (10 μg/ml) (Sigma-Aldrich, USA) for 24 h. The overexpression efficiency was detected under fluorescence microscope or by western blot. The cells were further treated with C-PC for 24 h and were divided into the following four groups: Vector group, Vector+C-PC group, ZDHHC5 group and ZDHHC5+C-PC group for functional experiments.

### Western blot analysis

After lysis with RIPA (Epizyme, Shanghai, China), the proteins were extracted and centrifuged. The supernatants were collected, and the total protein concentrations were measured using BCA (Epizyme, Shanghai, China) assay. Proteins were separated using SDS-PAGE (Epizyme, Shanghai, China) and transferred to PVDF membranes (Millipore, Germany) and blocked with protein free rapid blocking buffer for 1 h. The membranes were immunoblotted with the target primary antibodies at 4°C overnight. After washing with TBST, the membranes were treated with HRP-conjugated secondary antibody at room temperature for 1 h. Bands were visualized using chemiluminescence, and gray value analysis of the bands was performed using Image J software. The manufacturer and catalog of antibodies we used were listed in the Supplementary Table 1.

### Transcriptome sequencing

Total RNA was extracted using TRIzol reagent (New cell& Molecular Biotech, Suzhou, China)according to the manufacturer’s protocol. RNA purity and quantification were evaluated using NanoDrop 2000 spectrophotometer (Thermo Scientific, USA). RNA integrity was assessed using the Agilent 2100 Bioanalyzer (Agilent Technologies, Santa Clara, CA, USA). Then the libraries were constructed using TruSeq Stranded mRNA LT Sample Prep Kit (Illumina, San Diego, CA, USA) according to the manufacturer’s instructions. The transcriptome sequencing and analysis were conducted by OE Biotech Co. Ltd. in Shanghai, China. The libraries were sequenced on an Illumina HiSeq X Ten platform and 150 bp paired-end reads were generated for subsequent differentially expressed genes analysis.

### D-Gal-induced accelerated aging rat model

30 SD male rats (8 weeks old) (Charles River, Jinan, China) were used for animal experiments. 10 of them were injected with saline as the control group and 20 were subcutaneously injected with D-Gal (250 mg/kg) (Aladdin, Shanghai, China) for 40 days. After the establishment of the animal aging model, 10 mice were given saline by gavage as the D-Gal group. The remaining 10 were given C-PC (100 mg/kg) (Binmei Biotechnology, Taizhou, China) for 30 days as the D-Gal +C-PC group. Finally, the rats were sacrificed. BMSCs were extracted from bone marrows as reported previously [34]. The animal study was reviewed and approved by the Ethics Committee of Qingdao University.

### Enzyme-linked immunosorbent assay

Enzyme-linked immunosorbent assay (ELISA) kits (FANKEWEI, Shanghai, China) were used to detect the levels of interleukin-10 (IL-6) and transforming growth factor β (TGF-β) in MSCs. Samples were collected and tested according to the manufacturer's instructions. Analysis was performed within 15 min after adding the stop solution.

### Statistical analysis

Statistical analyses were performed using SPSS Statistics (Version: 25). All variables were described using mean ± standard deviation. Kruskal-Wallis test, a non-parametric alternative was used to calculate the comparisons between multiple groups. Further multiple comparisons were conducted by Bonferroni correction. The number of replicates and other statistical details were reported in the figure legends. P values less than 0.05 were considered statistically significant.

**Figure 1.**
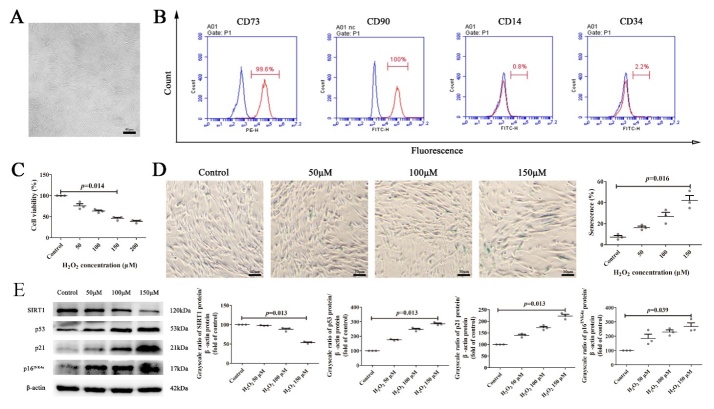
**The effects of H_2_O_2_ on Morphology, phenotype and senescence analysis of MSCs**. (**A**) The morphology of the 4th passage of MSCs. Scale bars, 40 μm. (**B**) Flow cytometry analysis of MSCs phenotype including CD73, CD90, CD14 and CD34. (**C**) Cell Counting Kit-8 (CCK-8) assay to detect the cell viability of MSCs in different groups. (**D**) Representative images of senescence-associated β-Galactosidase Staining and the quantification analysis of the percentage of senescence MSCs. Scale bars, 30 μm. (**E**) Western blot analysis of senescence related P53, P21, P16^INK4a^ and SIRT1 in MSCs and the quantification of western blot. Protein levels were normalized to β-actin. All results were presented as the mean ± SEM (n = 3). The P values were determined by Kruskal-Wallis test.

## RESULTS

### Isolation, characterization, and senescence model induction of MSCs

MSCs were isolated from human adipose tissue as reported previously [29]. Under microscope, MSCs show a spindle-shape, plastic adherent growth, and a vortex-like distribution (Fig. 1A). The phenotypic molecular analysis by flow cytometry showed that MSCs were positive for CD73^+^ and CD90^+^ expression with a positive rate above 95%. The expression of CD14^–^ and CD34^–^ in MSCs was negative with a positive rate below 3% (Fig. 1B). To investigate the roles of C-PC on MSCs senescence, we established a H_2_O_2_-induced MSCs senescence model. First, MSCs were exposed to different concentrations of H_2_O_2_ (0, 50, 100, 150 and 200 μM) for 2 h. The cell activity of MSCs decreased gradually with the increase of H_2_O_2_ concentration. Statistical results showed that 150 μM was the half-maximal inhibitory concentration (IC50) of H_2_O_2_ on MSCs (Fig. 1C). Next, several well-established senescence biomarkers were analyzed in these cells. Compared with the control group, the percentage of senescence-associated β-galactosidase (SA-β-gal) positive cells were significantly increased after exposure to oxidative stress. And the senescence rate of MSCs showed positive correlation to the concentration of H_2_O_2_ (Fig. 1D). The protein level of senescence marker, SIRT1 was decreased while the protein levels of P53, P21 and P16^INK4a^ were increased in a H_2_O_2_-concentration dependent manner (Fig. 1E). In conclusion, H_2_O_2_ can induce the senescence of MSCs and 150 μM was used as the optimal concentration for subsequent experiments.

**Figure 2.**
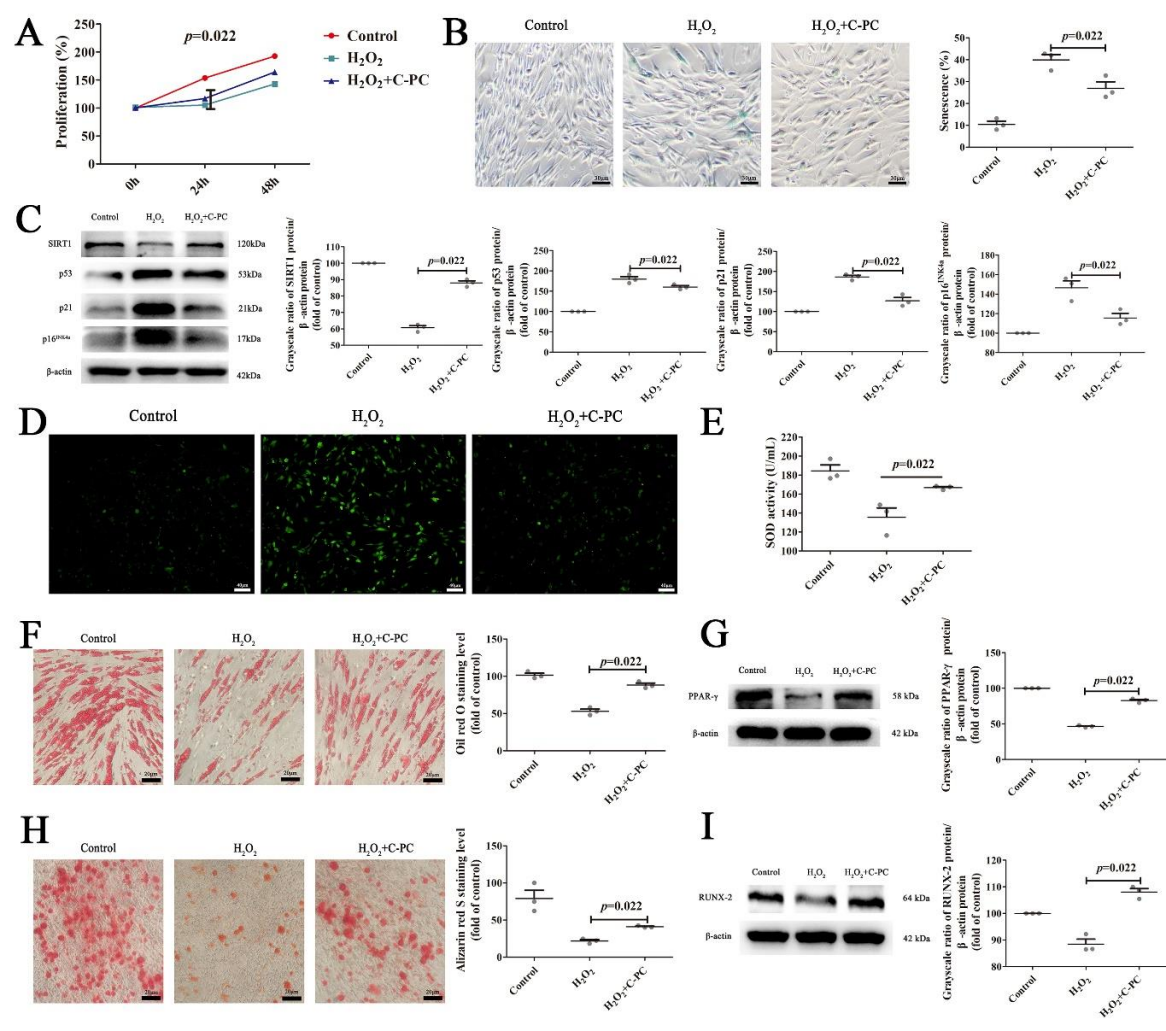
**C-PC inhibits senescence of MSCs *in vitro***. (**A**) CCK-8 assay to show the proliferation ability of senescent MSCs after C-PC treatment. (**B**) Representative images of β-Galactosidase staining and the quantification analysis of the percentage of senescence MSCs. Scale bars, 30 μm. (**C**) Western blot analysis of senescence related P53, P21, P16^INK4a^ and SIRT1 in MSCs and the quantification analysis of western blot. Protein levels were normalized to β-actin. (**D**) ROS staining of control, senescent and C-PC treated MSCs. Scale bars, 40 μm. (**E**) SOD activity assay of control, senescent and C-PC treated MSCs. (**F**) Representative images of Oil Red O staining and the quantification analysis of Oil Red O staining by Image-J software. Scale bars, 20 μm. (**G**) Western blot was performed to analyze the protein levels of adipogenic differentiation marker after treatment with C-PC. The quantification analysis of western blot was carried out to show the changes. Protein levels were normalized to β-actin. (**H**) Representative images of Alizarin Red S staining and the quantification analysis of Alizarin Red S staining by Image-J software. The red dots indicate the calcium deposits. Osteogenic differentiation potential was evaluated using calcium nodules. Scale bars, 20 μm. (**I**) The protein level of RUNX2, an osteogenic differentiation marker was analysed by Western blot. The quantification analysis of western blot was also performed to show the changes. Protein levels were normalized to β-actin. All results were expressed as the mean ± SEM (n = 3). The P values were determined by Kruskal-Wallis test.

**Figure 3.**
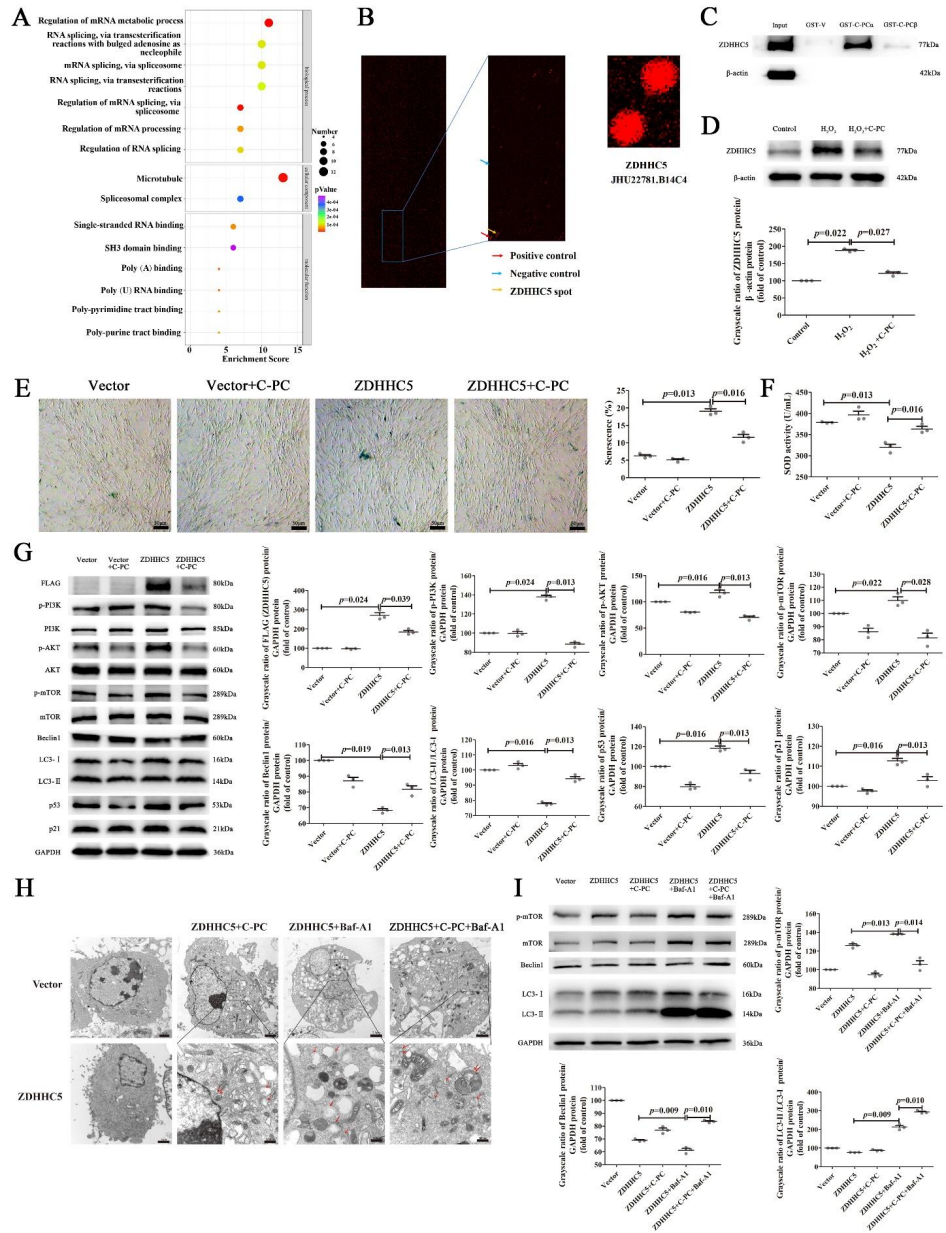
**C-PC attenuates MSC senescence by interfering with ZDHHC5-mediated autophagy**. (**A**) GO enrichment analysis showed that the differentially expressed genes were enriched in several biological processes. (**B**) Representative proteome chip results. Protein-protein interaction was detected (blue boxes) between C-PC and ZDHHC5 by red fluorescence. Representative images of protein chip showed positive control (red arrow), negative control (blue arrow) and ZDHHC5 spot (yellow arrow) on the enlarged images. (**C**) Pull down assay and the quantification analysis to show the interactions of α and β subunits of C-PC with ZDHHC5. (**D**) Western blot was performed to detect the protein levels of ZDHHC5 in different groups and the corresponding quantification analysis. β-actin was used as the internal control. (**E**) Representative images of β-Galactosidase staining and the quantification analysis of the percentage of senescence MSCs. Scale bars, 50 μm. (**F**) SOD activity assay of MSCs in different groups. (**G**)The protein levels of senescence, autophagy and PI3K/AKT/mTOR signaling pathway related proteins detected by Western blot and the corresponding quantification analysis. Protein levels were normalized to GAPDH. (**H**) Representative images of the observed autophagosomes by transmission electron microscope. One red arrow pointed to autophagosomes while two arrows pointed to autolysosome. Scale bars were shown in Figure. (**I**) Western blot analysis of the protein levels involved in autophagy and mTOR signaling after autophagy block by Baf-A1 (200 nM, 6 h) in MSCs. The quantification analysis of western blot was calculated by Image-J. Protein levels were normalized to GAPDH. Results were expressed as the mean ± SEM (n = 3). The P values were determined by Kruskal-Wallis test.

### C-PC inhibits senescence of MSCs in vitro

To detect the effect of C-PC on the senescence of MSCs, 250 μg/ml C-PC was added to the culture medium of senescent MSCs for 24h. Afterwards, the proliferation of MSCs was examined. The results showed that the proliferative activity of MSCs was increased after C-PC treatment. The statistical results showed that C-PC could restore the proliferation activity of senescent MSCs to a certain extent (Fig. 2A). The senescence rate of MSCs was markedly reduced as shown by SA-β-gal staining indicating that C-PC could improve the senescence of MSCs (Fig. 2B). Compared with the senescence group, C-PC group also increased the expression of SIRT1 and decreased the expression of P53, P21 and P16^INK4a^ (Fig. 2C). Since C-PC was reported to have anti-oxidation roles, the levels of ROS and SOD activities were detected. As expected, C-PC reduced the ROS levels (Fig. 2D) and increased SOD activities in senescent MSCs (Fig. 2E). These results indicate that C-PC has antioxidant capacity to repair H_2_O_2_-induced oxidative stress damage. Next, we examined the effects of C-PC on MSCs functions. MSCs were differentiated with adipogenic or osteogenic induction media, respectively. As shown by Oil Red O staining, more lipid droplets were present in C-PC-treated MSCs than senescent MSCs (Fig. 2F). Besides, the protein level of peroxisome proliferator-activated receptor γ (PPAR-γ), a specific transcription factor for adipocyte differentiation was also increased (Fig. 2G). Similarly, more calcium deposits were detected in C-PC-treated MSCs than senescent MSCs by Alizarin Red S staining (Fig. 2H). C-PC treatment also elevated the level of osteogenic transcription factor, RUNX-2 (Fig. 2I). These results showed that C-PC could enhance the adipogenic and osteogenic differentiation potential of MSCs. In summary, C-PC inhibited the senescence of MSCs *in vitro*.

### C-PC attenuates MSC senescence by interfering with ZDHHC5-mediated autophagy

To further illustrate the molecular mechanisms of C-PC in senescent MSCs, we performed a human proteome chip assay to screen the candidate proteins interacting with C-PC. Gene Ontology (GO) functional enrichment analysis showed the interacting candidate proteins were especially enriched in regulation of mRNA metabolic process and the cellular component such as microtube (Fig. 3A). Among all these candidates, ZDHHC5, a member of palmitoyltransferase family, was among the top hits with strong specificity (Fig. 3B). To verify the interaction between C-PC and ZDHHC5 and find the specific binding sites, the α and β subunit of C-PC were expressed and purified as described previously [19]. According to the results of pull-down assay, ZDHHC5 could bind to the α subunit of C-PC (Fig. 3C). Interestingly, the protein level of ZDHHC5 in senescent MSCs was increased (Fig. 3D). To assess the potential effect of ZDHHC5, MSCs were transfected with ZDHHC5 expressing-lentivirus. The senescence was aggravated in MSCs overexpressing ZDHHC5, which was shown by stronger SA-β-gal staining (Fig. 3E). A decrease in the activities of SOD was also detected (Fig. 3F). C-PC administration reversed all these results caused by ZDHHC5 overexpression. Since autophagy is involved in cell senescence, we wondered whether ZDHHC5 affected autophagy in MSCs. As expected, ZDHHC5 overexpression decreased the protein level of autophagy related Beclin1 and LC3. Autophagy related PI3K/AKT/mTOR signaling activation was also observed. What’s more, C-PC treatment reversed all the roles of ZDHHC5 in MSCs (Fig. 3G). Besides, the autophagosomes were observed in C-PC treated MSCs (Fig. 3H). The blockade of autophagy flux by bafilomycin A1 (Baf-A1) (200 nM, 6 h) was shown by accumulated LC3-II. C-PC administration increased the autophagy flux in ZDHHC5-overexpressed MSCs (Fig. 3I). These results showed that ZDHHC5 inhibited autophagy and caused senescence of MSCs. C-PC inhibited senescence of MSCs by reversing the roles of ZDHHC5.

**Figure 4.**
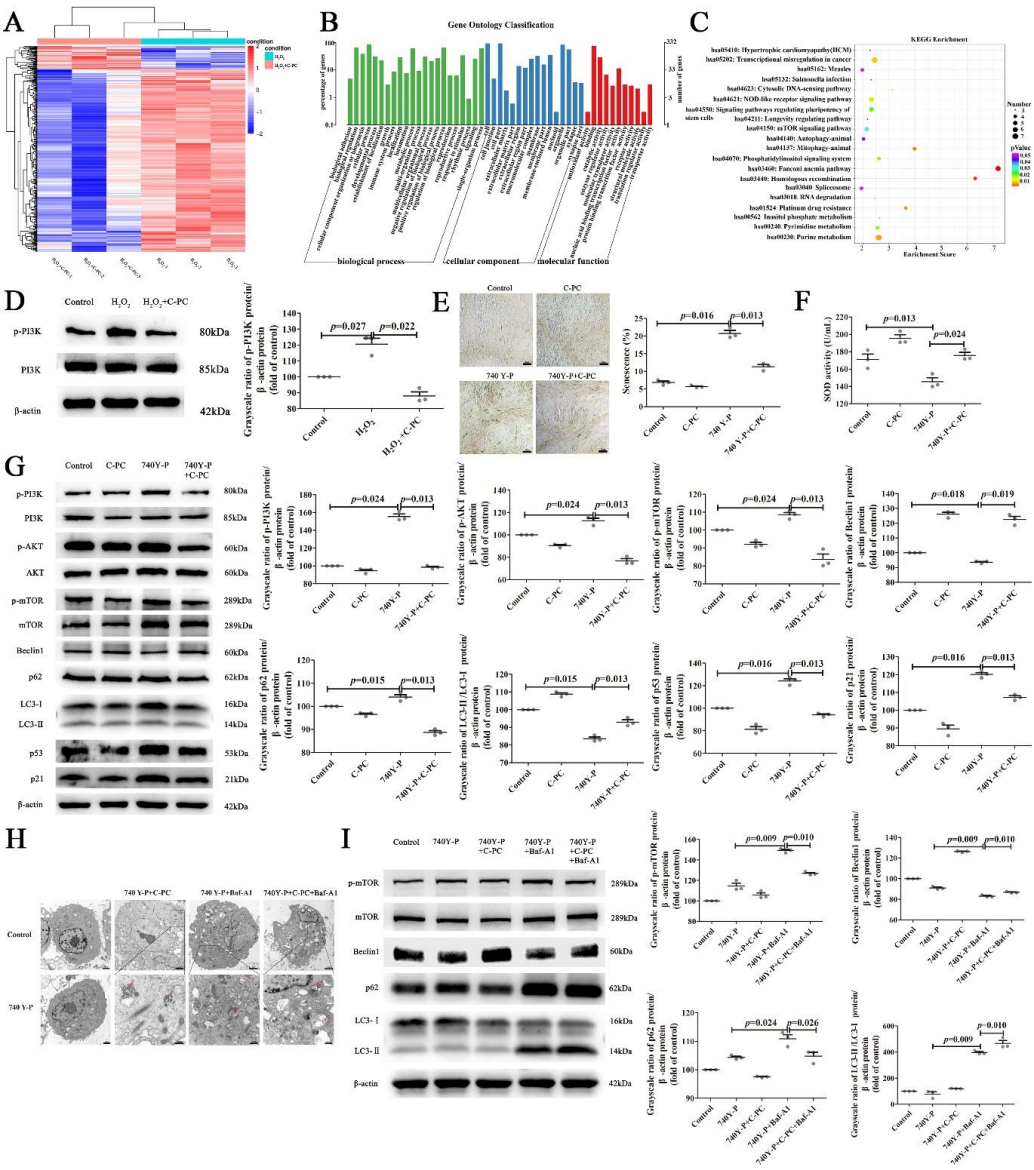
**C-PC inhibited MSCs senescence by downregulating PI3K/AKT/mTOR pathway**. (**A**) Transcriptome sequencing analysis. A heat map of differentially expressed genes in senescent and C-PC treated MSCs. (**B**) GO enrichment analysis to show the enriched biological processes of differentially expressed genes. (**C**) KEGG pathway analysis to show the signaling pathway which differentially expressed genes were enriched in. (**D**) The protein levels of p-PI3K by Western blot and the quantification analysis. Protein levels were normalized to β-actin. (**E**) Representative images of β-Galactosidase Staining and the quantification analysis by Image J software. Scale bars, 50μm. (**F**) SOD activity assay of MSCs in different groups. (**G**) Western blot and the quantification analysis of senescence, autophagy and PI3K/AKT/mTOR pathway related proteins. β-actin was used as the internal control. (**H**) Representative images of the observed autophagosomes by transmission electron microscope. One red arrow pointed to autophagosomes while two arrows pointed to autolysosome. Scale bars were shown in figure. (**I**) Western blot analysis of the protein levels involved in autophagy and mTOR signaling after autophagy block by Baf-A1 (200 nM, 6 h)in MSCs of different groups. β-actin was used as the loading control. Results were expressed as the mean ± SEM (n = 3). The P values were determined by Kruskal-Wallis test.

### C-PC inhibited MSCs senescence by downregulating PI3K/AKT/mTOR pathway

To further explore whether C-PC inhibits senescence by regulating autophagy and the underling signaling pathway, we systematically assessed the differentially expressed genes by transcriptome sequencing. The heat map analysis showed that there were significant gene expression differences between senescent MSCs group and the C-PC treated MSCs group (Fig. 4A). A total of 399 differentially expressed mRNA (absolute fold-change≥1.5; P-value≤0.05) was detected. Among them, 46 mRNAs were upregulated in MSCs treated with C-PC compared to the senescent group, while 353 mRNAs were downregulated. Next GO functional enrichment analysis was performed on these differentially expressed genes. A total of 49 terms were enriched including 22 biological processes, 16 cellular components and 11 molecular functions. Biological adhesion, biological regulation and cellular component organization were the top 3 enriched biological processes. Cell, cell junction and cell part were listed at the front of cellular components. Antioxidant activity, binding and catalytic activity were mostly enriched in molecular functions (Fig. 4B). Meanwhile, KEGG pathway enrichment analysis disclosed 20 signaling pathways such as mTOR signaling pathway, longevity regulating pathway, autophagy-animal, regulating pluripotency of stem cells and so on (Fig. 4C). PI3K is involved in multiple signaling pathways such as mTOR signaling pathway and autophagy-animal cascade, it also plays a role in regulating stem cell pluripotency and longevity. Therefore, we speculated that C-PC might inhibit MSCs senescence by PI3K/AKT/mTOR signaling pathway. Indeed, the level of p-PI3K was increased in senescent MSCs which were inhibited by C-PC (Fig. 4D). To further validate the regulatory mechanism of PI3K, normal MSCs were treated with PI3K activator, 740Y-P (20 μM) for 24 h. The senescence of MSCs was observed and C-PC attenuated the senescence caused by PI3K activation in MSCs (Fig. 4E). The activity of SOD from MSCs was also improved by C-PC in 740Y-P activated MSCs (Fig. 4F). Further research showed that C-PC reduced the levels of p-PI3K, p-AKT and p-mTOR, enhanced the autophagy level and therefore attenuated senescence of 740Y-P activated MSCs (Fig. 4G). In addition, the number of autophagosomes was increased in C-PC treated groups (Fig. 4H). Baf-A1 was used to block the autophagy flux. C-PC administration increased the autophagy flux in 740Y-P activated MSCs (Fig. 4I). These results showed that activation of PI3K promoted senescence of MSCs and C-PC inhibited senescence by inhibiting the autophagy related PI3K/AKT/mTOR pathway.

### C-PC attenuated MSCs senescence in vivo

**Figure 5.**
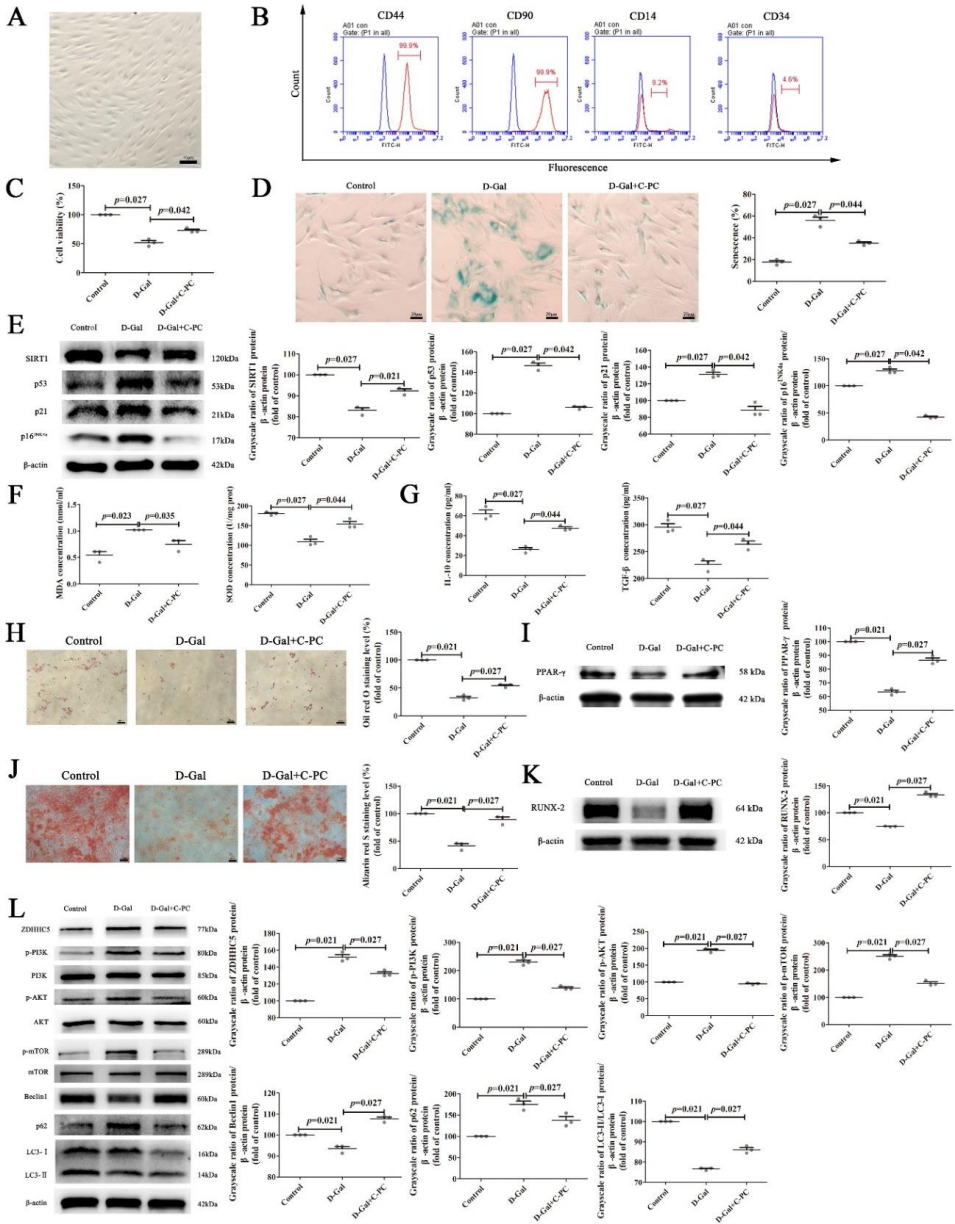
**C-PC attenuated MSCs senescence *in vivo***. (**A**) The morphology of the 4th passage of rat BMSCs. Scale bars, 40 μm. (**B**) Flow cytometry analysis of rat BMSCs immuno-phenotype including CD44, CD90, CD14 and CD34. (**C**) CCK-8 assay was performed to detect the viability of MSCs in different groups. (**D**) Representative images of β-Galactosidase Staining and the quantification analysis of the percentage of senescent BMSCs. Scale bars, 20 μm. (**E**) Western blot analysis of senescence related P53, P21, P16^INK4a^ and SIRT1 in different groups. Protein levels were normalized to β-actin. (**F**) The content detection of MDA in BMSCs and SOD activity detection of BMSCs. (**G**) Enzyme-linked immunosorbent assay (ELISA) was carried out to detect the levels of interleukin-10 (IL-10) and transforming growth factor β (TGF-β) in BMSC. (**H**) Representative images of Oil Red O staining and the quantification analysis of Oil Red O staining by Image-J software. Scale bars, 40 μm. (**I**) The protein level of PPAR-γ,an adipogenic differentiation marker was detected by Western blot. The quantification analysis of western blot was carried out to show the changes. Protein levels were normalized to β-actin. (**J**) Representative images of Alizarin Red S staining and the quantification analysis of Alizarin Red S staining by Image-J software. The red dots indicate the calcium deposits. Osteogenic differentiation potential was evaluated using calcium nodules. Scale bars, 40 μm. (**K**) The protein level of RUNX2, an osteogenic differentiation marker was analysed by Western blot. The quantification analysis of western blot was also performed to show the changes. Protein levels were normalized to β-actin. (**L**) Western blot and quantification analysis of the protein levels involved in autophagy, PI3K/AKT/mTOR signaling. β-actin was used as a loading control. Results were expressed as the mean ± SEM (n = 3). The P values were determined by Kruskal-Wallis test.

The therapeutic effects of C-PC on oxidative stress-induced MSCs senescence *in vitro* prompt us to investigate their role in D-Galactose (D-Gal) -induced accelerated aging rat model *in vivo*. Briefly, rats were induced to aging with subcutaneous injection with D-Gal (250 mg/kg) for 40 days, and then treated with C-PC (100 mg/kg) for 30 days by gavage [35]. Since bone marrow is one important source of MSCs and aging impairs proliferation, induces senescence and chondrogenic response of BMSCs while it has no negative effects on AMSCs [36] , we chose BMSCs to validate the therapeutic effects of C-PC *in vivo*. The isolation and cultivation of BMSCs were carried out as reported previously [37]. The morphology (Fig. 5A) and immunological phenotypes (Fig. 5B) validated its identity as BMSCs. We found that C-PC improved the viability (Fig. 5C) and hindered the senescence rate of BMSCs in aging rats (Fig. 5D). Compared with the aging group, C-PC administration increased the level of senescence-related protein SIRT1, while decreased the expression of P53, P21 and P16^INK4a^ in BMSCs (Fig.5E). These results indicated that C-PC could delay the senescence of BMSCs *in vivo*. C-PC are reported to exert anti-oxidation and anti-inflammation roles in aging models. In order to better study the therapeutic effect of C-PC on BMSCs in aging rats, we also detected the level of oxidative stress marker MDA and SOD activity in BMSCs. C-PC decreased the level of MDA and enhanced SOD activity from BMSCs (Fig. 5F). Besides, the levels of anti-inflammatory cytokines IL-10 and TGF-β were also elevated in BMSCs (Fig. 5G). BMSCs were differentiated with adipogenic or osteogenic induction media, respectively. As shown by Oil Red O staining, more lipid droplets were present in BMSCs of C-PC-treated aging rats (Fig. 5H). Besides, the protein level of PPAR-γ was also increased (Fig. 5I). Similarly, more calcium deposits were detected in BMSCs of C-PC-treated aging rats by Alizarin Red S staining (Fig. 5J). C-PC treatment also elevated the level of RUNX-2 (Fig. 5K). Finally, the autophagy and PI3K/AKT/mTOR signaling pathway related proteins were measured in BMSCs from different groups. The results showed that C-PC treatment also promoted autophagy by inhibiting the autophagy related PI3K/AKT/mTOR signaling pathway (Fig. 5L). The above data suggest C-PC could attenuate MSCs senescence *in vivo*.

## DISCUSSION

In this study, we aim to find a rational drug intervention to delay MSCs senescence. C-PC is a compound isolated from a variety of seaweeds such as *Spirulina* which is a well-known for its anti-aging effect [38]. C-PC has been reported to have multiple functions such as antitumor, antioxidation, anti-inflammatory and anti-aging roles [39]. So far, no research about the roles of C-PC on stem cell senescence has been conducted. Here, we systematically study the roles and mechanisms of C-PC on senescent MSCs. We demonstrated that C-PC could protect against H_2_O_2_-induced senescence of MSCs and improve their proliferation and differentiation. The therapeutic role of C-PC on preventing MSCs senescence was also observed in D-Gal-induced accelerated aging rat model *in viv*o. Our research is the first study, to our knowledge, to show that C-PC could inhibit MSCs senescence *in vitro* and *in vivo*.

Oxidative stress model constructed by H_2_O_2_ is often used in cell senescence research [40] and the current best practice to identify senescent cells are used in our study including the altered expression of senescence markers, positive staining for SA-β-gal and cell function abnormities. The altered levels of several proteins such as SIRT1, P53, P21 and P16^INK4a^ are typical characteristics of senescent cells [17, 41]. We also observed the increased expression of P53, P21 and P16^INK4a^ in our senescent MSCs model.

Many researchers use D-galactose-induced aging animals as an experimental model for aging research [16]. In this model, D-Gal is long-term supplied excessively which causes changes similar to natural aging [5]. Naturally aging model is a time-consuming process while D-Gal-induced aging is more preferred due to its easy application, shorter duration of study and higher survival rate of animals throughout the experimental period. We also chose D-Gal-induced aging to study the potential therapeutic roles of C-PC on senescent MSCs *in vivo*. Since oral administration of health care products is prevalent nowadays and therefore, we tested the roles of C-PC in aging rats by gavage.

MSCs are differentially influenced by aging. Some researchers found that BMSCs from aging animals had impaired proliferation, induced senescence and chondrogenic response, whereas muscle-derived stem cells and AMSCs exhibited no negative effects [36]. Thus, we chose MSCs from bone marrow to check the roles of C-PC *in vivo*. Someone may wonder how C-PC attenuates MSCs senescence through gavage. C-PC can be digested into peptides and absorbed into blood by intestine. They may carry out their potential roles as biological active peptides *in vivo*. Besides, many studies found C-PC decreased the inflammatory factors in serum of aging animals. The level of MDA and the activity of SOD in serum were also improved by C-PC [27]. The gut microbiota may also be involved in these inflammation diseases [42]. Altogether, it’s a complex process to delay senility *in vivo* for C-PC which needs further research in future.

Next, we focused on the mechanism of C-PC on MSCs senescence. We performed a human proteome chip assay to screen out the interacting proteins. Currently, the HuProt™ Human Proteome Chip is the highest throughput human protein chip, covering approximately 20,000 full-length human proteins. ZDHHC5, a member of palmitoyltransferases family, was screened out by GO functional enrichment analysis and chip scanning data analysis. Molecular docking and other experiments are needed in future to find the specific interacting sites between C-PC and ZDHHC5. Besides, we found that C-PC could also down-regulate the expression of ZDHHC5 which also needs further work.

ZDHHC5, one of 23 ZDHHC protein acyltransferase family members, was found to localize on the plasma membrane, endoplasmic reticulum, and the Golgi apparatus. ZDHHC5 could catalyze protein palmitoylation as a dynamic and reversible post-translational modification [43, 44]. ZDHHC5 is highly expressed in a variety of embryonic cells including neural progenitor cells with an undifferentiated state, but is rapidly degraded during differentiation [45]. It is also upregulated in glioma stem cells (GSC) and contributes to the tumor development and progression [44, 45]. These results revealed that ZDHHC5 played important roles in stem cells. Therefor we chose ZDHHC5 for further study.

Back to MSCs senescence, what’s the relationship between ZDHHC5 and MSCs senescence?How did ZDHHC5 affect MSCs senescence? To answer these questions, we overexpressed ZDHHC5 in MSCs. We noticed that ZDHHC5 was closely related to MSCs senescence and autophagy which might be involved in this process. Autophagy is a degradation mechanism that attenuates senescence of cells through degrading long-lived proteins, pathogens, damaged DNA elements, and damaged organelles. It is activated especially during metabolic stress conditions [6]. Young quiescent MSCs have a constitutive autophagy activity that diminishes with aging. The impairment occurs in old cells which is related to the lack of autophagosome formation capacity [46]. The microtubule-associated protein 1 light chain 3 (LC3) is a key regulator of autophagy. LC3 controls the growth of autophagic membranes, the recognition of autophagic cargoes and the fusion of autophagosomes with lysosomes in the autophagic pathway [47].

**Figure 6.**
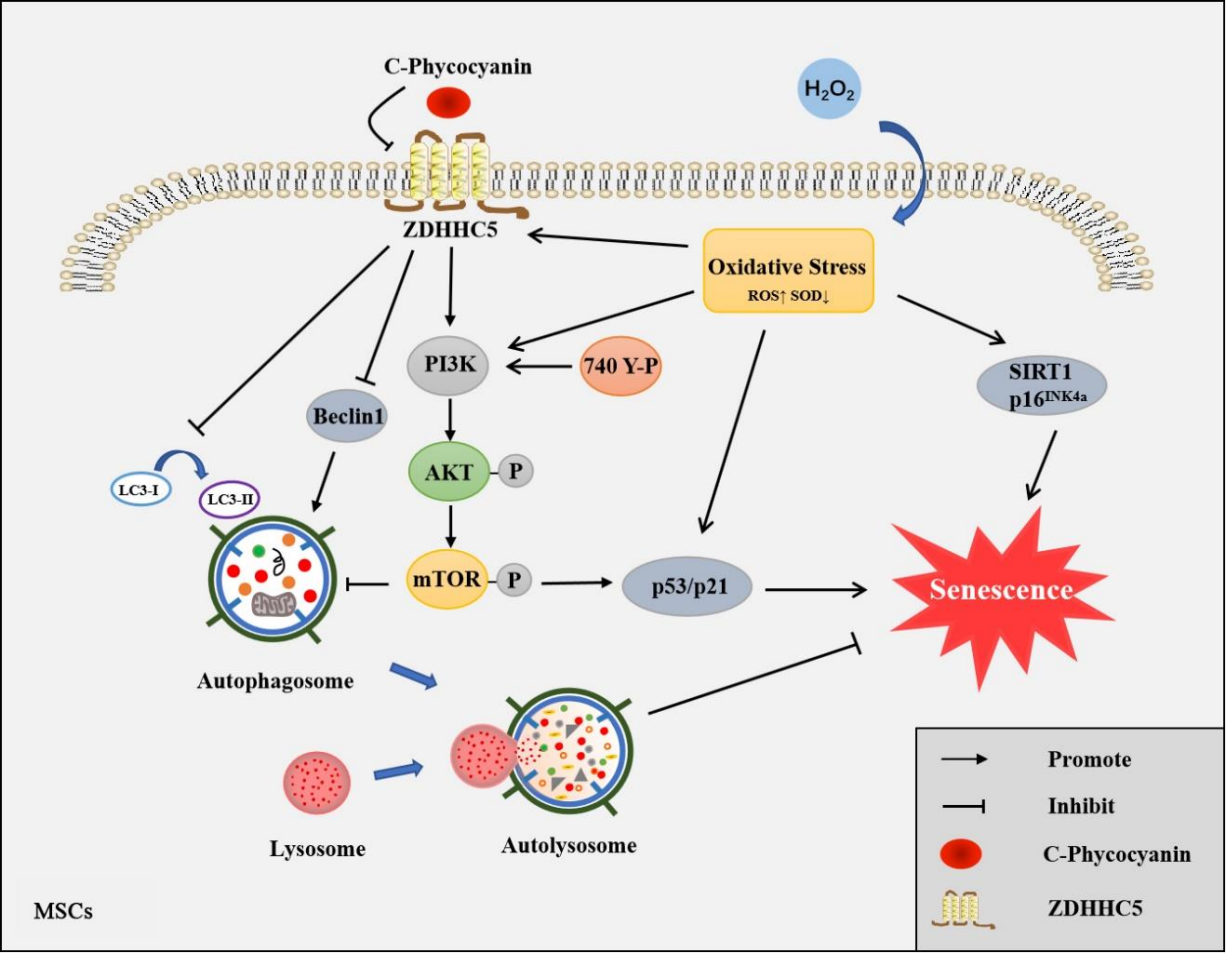
**C-PC ameliorates the senescence of MSCs through ZDHHC5 mediated autophagy via PI3K/AKT/mTOR pathway**. Oxidative stress induced senescence of MSCs and increased the level of ZDHHC5 *in vitro*. ZDHHC5 activated PI3K/AKT/mTOR signaling and inhibited autophagy which finally promoted MSCs senescence. C-PC downregulated and reversed all the roles of ZDHHC5 on MSCs senescence.

In order to demonstrate the molecular mechanisms, we performed transcriptome sequencing of C-PC treated MSCs and senescent MSCs meantime. Based on GO and KEGG pathway enrichment analysis, we selected the differentially expressed key protein PI3K and its mediated PI3K/AKT/mTOR autophagy pathway. Activation of PI3K/AKT/mTOR pathway not only contributed to the regulation of apoptosis, oxidative stress and autophagy but is also considered central to aging. mTOR activation in various tissues during aging is considered a major driving force [48, 49]. Additionally, studies have also reported the regulatory role of ZDHHC5 in controlling target protein stability through the crosstalk with autophagy, and further revealed the association between S-palmitoylation-based autophagy [50]. All these results and references proved that autophagy-related PI3K/AKT/mTOR pathway was important to attenuate MSCs senescence.

Next question is about the relationship between ZDHHC5 and PI3K/AKT/mTOR pathway. How did ZDHHC5 affect PI3K/AKT/mTOR pathway? The mechanism is still unclear since there are few studies on ZDHHC5 at present and most of them are only reported to be related to cancer. Protein post-translational modifications (PTM) are hotspots in recent years, and it still has a long way to go to illustrate the roles of ZDHHC5 in MSCs senescence.

In summary, our study demonstrated for the first time that C-PC ameliorates the senescence of MSCs through ZDHHC5 mediated autophagy via PI3K/AKT/mTOR pathway (Fig. 6). Our findings suggested that autophagy and PI3K/AKT/mTOR pathway may be potential targets for the anti-senescence studies of MSCs.

## Supplementary Materials

The Supplementary data can be found online at: www.aginganddisease.org/EN/10.14336/AD.2023.0121.
