# Supplementary material for: C-Phycocyanin Ameliorates the Senescence of Mesenchymal Stem Cells through ZDHHC5-Mediated Autophagy via PI3K/AKT/mTOR Pathway
Source: Aging Dis. 2023 Aug 1;14(4):1425–40. doi: 10.14336/AD.2023.0121 (PMC10389819; doi:10.14336/AD.2023.0121)
Supplement: Supplementary file 1 — The Supplementary data can be found online at: www.aginganddisease.org/EN/10.14336/AD.2023.0121. [file AD-14-4-1425-s.pdf]

SUPPLEMENTARY DATA

C-Phycocyanin Ameliorates the Senescence of Mesenchymal Stem Cells through ZDHHC5-Mediated Autophagy *via* PI3K/AKT/mTOR Pathway

Guoxiang Liu, Xiaoxia Li, Fanghao Yang, Jingyu Qi, Lipeng Shang, Huhu Zhang, Shuang Li, Fenghua Xu, Lingne Li, Huaxin Yu, Yang Li, Xiaolei Dong, Qinghang Song, Feng Zhu, Guang Chen, Can Cao, Liangqian Jiang, Junzhe Su, Lina Yang, Xiaohui Xu, Zhe Zhang, Robert Chunhua Zhao, Bing Li

© 2023. Liu G et al. Published online at https://www.aginganddisease.org/EN/10.14336/AD.2023.0121

SUPPLEMENTARY DATA

Supplementary Table 1. The manufacturer and catalog of antibodies

Antibody Name	Manufacturer	Catalog number
CD73	Biolegend	344016
CD90	Biolegend	328108
CD14	BD Biosciences	557153
CD34	BD Biosciences	560942
isotype control (Mouse IgG2a, κ Isotype)	BD Biosciences	563809
CD44	Biolegend	203906
CD90	Biolegend	206105
CD14	BOSTER	A01818
CD34	BOSTER	BA0532
FITC-labeled Goat anti-rabbit secondary antibody	BOSTER	BA1105
HRP-labeled Goat Anti-Mouse IgG	Epizyme	LF101
HRP-labeled Goat Anti-Rabbit IgG	Epizyme	LF102
Flag Flag	ZEN BIO	384091
β-actin	ZEN BIO	700068
ZDHHC5	Proteintech	21324-1-AP
P53	Proteintech	10442-1-AP
P21	ABclonal	A2691
p-PI3K (Y607)	Abcam	ab182651
PI3K	Abcam	ab191606
SIRT1	Abcam	ab189494
$P16^{INK4a}$	Abcam	ab211542
PPAR-γ	Abcam	ab272718
RUNX-2	Abcam	Ab236639
p-AKT (Ser473）	Cell Signaling Technology	4060
AKT	Cell Signaling Technology	4691
p-mTOR (Ser2481）	Cell Signaling Technology	381548
mTOR	Cell Signaling Technology	380411
Beclin1	ZEN BIO	R22856
LC3	Proteintech	14600-1-AP

© 2023. Liu G et al. Published online at https://www.aginganddisease.org/EN/10.14336/AD.2023.0121
